# Longitudinal DLQI trajectory patterns and early risk updating in telepharmacy-monitored psoriasis

**DOI:** 10.3389/fdgth.2026.1961097

**Published:** 2026-10-26

**Authors:** Gabriel Mercadal-Orfila, Sergio Salvador-Pareja, Jorge Mateo Sotos, Ana M. Torres Aranda, Manuel Pinteño-Blanco, María Antonia Maestre-Fullana, Santiago Ríos, Pedro Tauler Riera, Salvador Herrera-Pérez

**Affiliations:** 1 Pharmacy Department, Hospital General Mateu Orfila, IB-Salut, Mahón, Menorca, Balearic Islands, Spain; 2 Research Group on Evidence, Lifestyles and Health, IUNICS, University of the Balearic Islands, Palma, Balearic Islands, Spain; 3 Pharmacy Department, Hospital Comarcal D'Inca, Inca, Balearic Islands, Spain; 4 Doctoral Programme in Translational Research in Public Health and High Prevalence Diseases, University of the Balearic Islands (UIB), Palma, Balearic Islands, Spain; 5 Medical Analysis Expert Group, Institute of Technology, University of Castilla-La Mancha, Cuenca, Spain; 6 Medical Analysis Expert Group, Castilla-La Mancha Institute of Health Research (IDISCAM), Toledo, Spain; 7 Pharmacy Department, Hospital Manacor, Manacor, Balearic Islands, Spain; 8 Departament de Genètica, Microbiologia I Estadística, Facultat de Biologia, Universitat de Barcelona, Barcelona, Spain; 9 Fundamental Biology and Health Sciences, Universidad de las Islas Baleares, Health Research Institute of the Balearic Islands (IdISBa), Santiago de Compostela, Spain; 10 Faculty of Health Sciences, International University of Valencia, Valencia, Spain; 11 Faculty of Health Science, Technology and Management, Universidad Intercontinental de la Empresa (UIE), Santiago de Compostela, Spain

**Keywords:** dermatology life quality index, machine learning, NAVETA, patient-reported outcomes, psoriasis, telepharmacy, trajectory-pattern analysis

## Abstract

**Background:**

Telepharmacy platforms capture longitudinal patient-reported health-related quality-of-life (HRQoL) data in psoriasis, but reports have emphasized average improvement, which may conceal a minority with persistent burden. Whether machine learning adds value beyond baseline severity, and whether the first follow-up sharpens risk estimation, remain unclear.

**Objective:**

To characterize 12-month Dermatology Life Quality Index (DLQI) trajectories in a telepharmacy psoriasis cohort, to identify a persistently impaired minority through trajectory-pattern analysis, and to determine where the added value of longitudinal monitoring lies.

**Methods:**

After curation of the NAVETA telepharmacy dataset, the analytic cohort consisted of 219 patients contributing a total of 831 observations. As the primary analysis, unsupervised Ward clustering of DLQI trajectories (a complete-case subset of 133 of the 219 patients with complete baseline-to-month-6 DLQI) identified exploratory trajectory patterns. A leakage-aware benchmark compared a null rule, a one-predictor baseline-DLQI logistic model, multivariable logistic regression and four ensemble learners for 6-month clinically meaningful improvement (≥4-point reduction) across 100 repeated stratified 80/20 splits, with calibration. For 6-month remission (DLQI ≤ 1), a baseline-DLQI-only comparator and a month-1 updating model were evaluated in the same dynamic cohort.

**Results:**

Mean DLQI improved from 7.78 to 2.89 over 12 months. Three exploratory trajectory patterns were identified—low-burden early control (*n* = 90), high-burden responder (*n* = 20) and persistent burden (*n* = 23; 17.3%); the retained three-cluster solution showed a silhouette of 0.43, and the last pattern remained impaired at 12 months (mean DLQI 7.31 vs. 1.91 and 1.80; *p* = 0.009). For improvement, a one-predictor baseline-DLQI logistic model matched the leading ensembles (AUROC 0.923 vs. XGBoost 0.903), and feature-attribution analysis confirmed the dominance of baseline DLQI. For DLQI remission, the baseline-DLQI-only model reached AUROC 0.641, whereas the month-1 DLQI updating model reached AUROC 0.753 with good calibration (intercept −0.02, slope 1.04, ECE 0.021).

**Conclusions:**

A favourable average conceals a clinically important minority with persistent HRQoL burden that trajectory-pattern analysis can identify. For outcomes anchored to baseline severity, machine learning adds little beyond a simple baseline-DLQI model; the added value of telepharmacy may instead emerge from longitudinal information, with the first follow-up providing an early, well-calibrated update to individual risk. External validation is required.

## Introduction

Psoriasis is a chronic, immune-mediated inflammatory skin disease that affects approximately 2% to 3% of the global population and imposes a burden that extends well beyond visible cutaneous lesions (1). In moderate-to-severe disease, this burden routinely involves work participation, social functioning, daily activities, emotional wellbeing and treatment satisfaction. For this reason, the success of contemporary treatment is judged not only by skin clearance but by whether the aspects of daily life that matter to patients improve. Dermatology-specific patient-reported outcome measures (PROMs) have consequently become a core component of psoriasis care, and among them the Dermatology Life Quality Index (DLQI) remains one of the most widely used instruments for capturing health-related quality of life (HRQoL). Its brevity, its validated 0-to-30 scoring and its interpretive bands—most notably the score of 0 or 1 that denotes no effect of disease on a patient's life—make it particularly suitable for repeated, longitudinal monitoring (2, 3), and systematic reviews continue to support its central role among psoriasis outcome measures (4).

In parallel, the integration of electronic PROMs (ePROMs) into telepharmacy pathways has expanded rapidly. The telepharmacy model endorsed by the Spanish Society of Hospital Pharmacy explicitly encompasses pharmacotherapeutic follow-up, remote outcome monitoring and digital communication as components of specialized pharmaceutical care (5). The NAVETA initiative has operationalized this model at scale, building a multicenter, value-based infrastructure for the systematic collection of ePROMs and ePREMs (electronic patient-reported experience measures) across chronic conditions (6). In psoriasis specifically, several NAVETA-dataset-based studies have already documented clinically relevant improvements in DLQI and psoriasis-specific symptom burden over 6 and 12 months of telepharmacy follow-up (7, 8). The feasibility and the average benefit of telepharmacy-supported monitoring in this population are therefore reasonably well established.

What remains unexamined is what those average figures may conceal. Mean improvement is clinically useful, but it is a summary measure that can mask subgroups with distinct trajectories over time. Two patients may contribute identically to the same average change while following opposite paths: one may improve quickly to minimal residual burden, whereas another may remain persistently impaired despite treatment initiation or switching. For precision follow-up, these individual trajectories matter far more than the cohort mean. Because a telepharmacy platform repeatedly captures the same ePROMs, these longitudinal data can support trajectory-pattern analysis: the data-driven identification of clinically meaningful patterns from routinely collected digital outcomes.

Machine-learning applications in psoriasis have concentrated on image-based diagnosis, lesion segmentation, automated severity scoring, biomarker discovery and prediction of biologic-treatment response (9, 10), rather than on the longitudinal patient-reported data generated during remote follow-up. Machine-learning studies in clinical prediction are susceptible to overstated performance when they do not include simple comparator models, permit outcome-related leakage, or fail to report calibration. These concerns may be particularly relevant for ePROMs-based datasets, in which baseline burden and follow-up timing can strongly influence apparent change. Accordingly, current reporting guidance emphasizes these safeguards, and existing evidence suggests that more complex algorithms do not consistently outperform well-specified regression models in clinical settings. Any claim that machine learning adds incremental value to telepharmacy outcome data should therefore be evaluated against parsimonious baselines rather than assumed (11, 12).

Two gaps follow from this. First, despite the effectiveness of modern systemic and biologic therapies, a clinically relevant residual burden persists for some patients, and real-world data have linked this residual impairment to factors such as obesity and therapeutic complexity (13, 14); however, to the best of our knowledge, no analysis has determined whether it is possible to identify distinct health-related quality of life (HRQoL) trajectory patterns, including a minority with persistent HRQoL burden, based on longitudinal DLQI data. Second, if such a minority exists, it is unknown how early it can be recognized: whether the information needed to separate patients converging toward recovery from those drifting into sustained burden is already available after the first month of follow-up, or whether it requires prolonged observation. The clinical value of telepharmacy would be considerably greater if risk could be updated at the first reassessment rather than only in retrospect.

Accordingly, we designed a secondary analysis of the NAVETA-based psoriasis cohort structured around a single clinical question: whether the favourable average conceals a clinically important minority, and where the information needed to act on that minority actually resides. As the primary analysis, we characterized the 12-month DLQI trajectory and used unsupervised trajectory-pattern analysis to test whether a persistently impaired subgroup could be identified beneath the cohort mean. As a supporting analysis, we then benchmarked machine-learning against parsimonious models for an outcome that is, by construction, tied to baseline DLQI, in order to establish how much complex modelling adds when the endpoint is largely determined by initial severity. Finally, we tested whether incorporating the first follow-up measurement, the earliest longitudinal signal available through telepharmacy, updates individual risk. Together, these steps address not only whether outcomes can be predicted but where the added value of longitudinal telepharmacy monitoring lies. We hypothesized that a persistently impaired minority would be identifiable from longitudinal DLQI trajectories, and that the first follow-up measurement would improve individual risk estimation beyond baseline information.

## Materials and methods

### Study design and data source

We conducted a retrospective secondary analysis of an anonymized psoriasis dataset from the NAVETA telepharmacy programme, with data collected in public hospitals of the Spanish healthcare system. The NAVETA infrastructure and its parent psoriasis cohorts have been described previously (6–8). Eligible patients were adults (aged ≥18 years) with moderate-to-severe psoriasis followed with protocolized ePROM collection. Every predictive task was restricted to variables available at the intended time of prediction, to avoid temporal information leakage. The study was reported in accordance with the TRIPOD + AI statement for clinical prediction models developed with regression or machine-learning methods (12). Data were collected across 20 participating hospital pharmacy centres, with DLQI responses recorded between December 2021 and March 2026 (Supplementary Table S2). The NAVETA programme records the medication dispensed to each patient and the treatment line (including biologic-naïve status and switches between lines) in its dispensing records; in the curated, anonymized research export analysed here, however, the current-medication field was available for only about one third of patients and the treatment-line field was almost entirely missing (Supplementary Table S4), so neither was used in any analysis. The implications of this exclusion are addressed in the Limitations. The completed TRIPOD + AI checklist is provided as Supplementary material.

### Cohort reconstruction

Questionnaire sessions were organized into baseline-anchored follow-up stages, each defined by taking a recorded baseline visit as an inception point and attaching subsequent follow-up records until the next baseline visit or the end of available follow-up. Because a subset of patients contributed more than one follow-up stage, all analyses were restricted to the first follow-up stage per patient to preclude within-person dependence. Here, baseline denotes the index ePROM assessment that opens a monitoring stage rather than a treatment-initiation timepoint, and a treatment switch or restart within a stage does not open a new stage. Each patient therefore contributes a single stage to the supervised and clustering analyses; only the longitudinal mixed-effects model uses repeated measures, for which within-patient correlation was modelled with a per-patient random intercept. Cohort assembly and the size of every analytic subset are detailed in Supplementary Table S1. The DLQI outcome was not imputed at any timepoint: because the DLQI trajectory was itself the object of the unsupervised analysis, imputing the values that define the clustering would impose model-based structure on the outcome under study. Missingness was assumed to be at most missing-at-random conditional on observed baseline characteristics, an assumption that cannot be verified. Each analysis then used a complete-case subset defined by the timepoints it required: baseline through month 6 for trajectory-pattern analysis, baseline and month 6 for clinically meaningful improvement, baseline, month 1 and month 6 for dynamic remission updating, and baseline and month 12 for the descriptive 12-month summary.

### HRQoL measure and study endpoints

The DLQI total score was used as the canonical HRQoL outcome: a validated 0–30 index of the impact of skin disease on quality of life, in which higher scores indicate greater impairment (2). The primary aim was the identification of a persistent HRQoL-burden trajectory pattern and the assessment of its durability at 12 months. Secondary aims addressed the prediction of two established DLQI endpoints: 6-month clinically meaningful improvement, defined *a priori* as a reduction of at least 4 points from baseline (the minimal clinically important difference, MCID, for the DLQI, and the standard responder definition in psoriasis trials) (2, 15) and 6-month remission, defined as a DLQI of 1 or less, corresponding to no effect of disease on the patient's life. Clinically meaningful improvement, being defined relative to baseline, was used for the baseline benchmark because it is the natural setting in which to test whether machine learning adds value beyond baseline severity, whereas the more demanding DLQI remission was used for the month-1 updating analysis because it is the clinically decisive state for which early longitudinal information is most relevant. Throughout this article, remission refers to DLQI remission (DLQI ≤ 1), not to skin clearance.

### Candidate predictors and preprocessing

Baseline candidate predictors reflected information plausibly available at programme entry: baseline DLQI, sex, age range, BMI range, physical activity, balanced diet, patient-association membership, marital status, education level, employment status, living situation, alcohol use, tobacco use, caffeine use, other substance use and reported drug allergy. In the primary 6-month improvement cohort (*n* = 168), missingness was low for most covariates (0%–3.6% for sex, age range, BMI, patient-association membership, marital status, education level, employment status, living situation, alcohol use, other substance use and drug allergy) and moderate for physical activity, balanced diet, tobacco use and caffeine use (24.4% each). Age at smoking initiation, diagnosis date, treatment line and medication-level fields were not used in the primary models because of high missingness or limited reliability (51.2%, 73.2%, 99.4% and 73.2%, respectively; Supplementary Table S4). The exact model variable sets were as follows: the null rule and one-predictor model used baseline DLQI only; the multivariable logistic and ensemble models used baseline DLQI plus the baseline covariate set; the context-only sensitivity model used the same baseline covariate set after removing baseline DLQI and all DLQI-derived quantities; and the dynamic remission analysis used a baseline-DLQI-only comparator and a month-1 update.

Within every resampling partition, numeric predictors were median-imputed and standardized and categorical predictors were imputed with the most frequent category and one-hot encoded; all preprocessing was estimated on training data only. The DLQI outcome and the follow-up DLQI values required to define each endpoint were never imputed.

### Unsupervised trajectory-pattern analysis

Phenotypes were derived in the complete-trajectory cohort from the raw four-dimensional DLQI vectors comprising the baseline, month-1, month-3 and month-6 values. The objective was pragmatic and clinical: to obtain a small number of interpretable follow-up patterns to inform telepharmacy monitoring rather than to infer latent population classes; raw scores were retained rather than standardized because absolute burden, and not only trajectory shape, is clinically meaningful here, and we acknowledge that this makes the resulting groups sensitive to overall burden level as well as to shape. In particular, clustering raw DLQI values with Euclidean distance ties the resulting groups partly to baseline severity, a deliberate trade-off accepted because absolute burden is itself clinically meaningful. We used hierarchical agglomerative clustering with Ward linkage and Euclidean distance, which requires no distributional assumptions and yields a deterministic, fully reproducible solution.

Model-based longitudinal approaches (group-based trajectory modelling, latent class growth analysis, latent class mixed models and growth mixture models) were not adopted because they target latent-class inference and require larger samples than were available for stable estimation of small subgroups. Two- to five-cluster solutions were compared on internal validity (the silhouette coefficient) and clinical interpretability.

The two-cluster solution yielded the highest silhouette but merged two clinically distinct high-baseline patterns, rapid responders and patients with sustained impairment, whereas the four- and five-cluster solutions split the high-burden segment into small, unstable groups. The three-cluster solution was therefore retained as an explicitly analyst-informed, clinically motivated compromise rather than as the internally optimal partition, and its stability was assessed by bootstrap resampling with the adjusted Rand index. Given the moderate internal separation of the retained solution, the patterns were interpreted as overlapping clinical groupings rather than discrete entities.

### Benchmarking machine learning against parsimonious models

The prediction of 6-month clinically meaningful improvement was framed as a comparative benchmark rather than as the development of a single model. Because the endpoint is defined relative to baseline DLQI, a reduction of at least 4 points, baseline burden is, by construction, strongly related to the capacity for meaningful change, and a high apparent accuracy could arise simply from re-expressing baseline severity. To measure how much, if anything, machine learning adds beyond this dominant clinical variable, we arranged seven models along a ladder of increasing complexity and evaluated all of them under an identical protocol. The simplest was a deterministic clinical null rule that predicted improvement whenever baseline DLQI was 4 or greater. Because this deterministic rule yields a single ROC operating point, its AUROC equals the average of sensitivity and specificity and therefore coincides with its balanced accuracy. Next was a one-predictor logistic regression using baseline DLQI as the sole, continuous input, which served as the principal parsimonious reference against which the remaining models were judged. A multivariable logistic regression then added the full baseline covariate set, and four tree-based ensemble learners, RandomForest, ExtraTrees, XGBoost and LightGBM, provided flexible, non-linear alternatives able to capture interactions among predictors.

Internal validation used repeated stratified holdout resampling: in each of 100 repetitions, 80% of patients were assigned to training and 20% to an held-out test partition. On the held-out sets, discrimination, ranking performance and probabilistic accuracy were summarized as the mean ± standard deviation of the area under the receiver operating characteristic curve (AUROC), the area under the precision–recall curve (AUPRC), balanced accuracy and the Brier score across the 100 repetitions. Repeated holdout was preferred over a single split to stabilize estimates in a modest cohort; because it does not yield a formal optimism-adjusted estimate, the reported means represent internally validated test performance rather than optimism-corrected performance. Throughout, ±values denote the standard deviation across the 100 repeated validation partitions, not a confidence interval. Repeated holdout also ensured that all models were compared under identical test partitions, prioritizing fair comparison over final-model optimization. No hyperparameter tuning was performed: all models were fitted with hyperparameters prespecified before resampling (the default configurations of scikit-learn 1.8.0, XGBoost 3.1.3 and LightGBM 4.6.0), and no search over held-out data was undertaken; where algorithm-level randomization was present, the random seed was varied by repetition. The benchmark therefore estimates out-of-sample performance for prespecified modelling pipelines rather than tuned maximum performance. Two prespecified sensitivity analyses further probed the source of the predictive signal. First, the benchmark was repeated in the subgroup of patients with a baseline DLQI of 4 or greater, thereby reducing the deterministic component of the endpoint. Second, a context-only model excluded baseline DLQI and every DLQI-derived quantity, so that any remaining discrimination could be attributed to non-DLQI information.

### Dynamic month-1 risk updating

Because remission may depend more on early evolution than on baseline status, a dynamic updating analysis was built for 6-month remission in the cohort with baseline, month-1 and month-6 DLQI. Month 1 was chosen because it represents the earliest clinically actionable reassessment. Under the same 100 repeated stratified 80/20 design used for the improvement benchmark, three models were compared. The baseline-only logistic comparator used baseline DLQI as the sole continuous predictor, whereas the parsimonious month-1 updating model used month-1 DLQI as the sole continuous predictor. Baseline DLQI, month-1 DLQI and their difference were not entered simultaneously in any logistic model because of collinearity. The RandomForest update was broader and included early longitudinal DLQI information together with the available baseline sociodemographic, lifestyle and treatment-context covariates. Because the remission endpoint is measured at a genuinely later timepoint than the month-1 predictor and is not derived from any clustering step, the month-1 update does not introduce circularity.

### Model explainability and calibration

XGBoost was retained as the reference ensemble model for interpretation because it combined near-top discrimination with the strongest overall balance of ranking precision, balanced classification and probabilistic accuracy in the improvement benchmark. Model explanation used SHAP (SHapley Additive exPlanations) values summarized as mean absolute importance. Calibration was evaluated from out-of-sample probabilities generated during the repeated holdout procedure: for each patient, the predictions obtained while that patient was in a test set were averaged, and the resulting per-patient probabilities were summarized by the calibration intercept, the calibration slope and the quantile-binned expected calibration error (ECE).

### Statistical analysis

Longitudinal change in DLQI was modelled with a linear mixed-effects model in which DLQI was the dependent variable, follow-up visit was entered as a categorical fixed effect with baseline as the reference category, and a random intercept was fitted for each patient to account for within-patient correlation. All available repeated observations contributed, so patients with incomplete follow-up were retained. Models were fitted by restricted maximum likelihood (REML). A random-slope specification for time was explored as a sensitivity analysis, but the random-intercept model was retained as the primary specification for parsimony and stability in a dataset with unequal follow-up. Residual assumptions were assessed graphically with quantile–quantile and residual-vs.-fitted plots. Coefficients are reported with 95% confidence intervals and *p* values.

Differences across trajectory patterns were examined with Kruskal–Wallis tests for continuous variables and chi-square tests for categorical variables. As an exploratory check of durability, the 6-month patterns were extended to month 12 in the subset of clustered patients with an available month-12 measurement, using the same non-parametric tests. Proportions are reported with Wilson 95% confidence intervals. Between-pattern comparisons (Table 1 and Supplementary Table S3) are exploratory and were not corrected for multiple testing. No formal sample-size calculation was performed: all patients meeting the data-availability criteria were analysed, and the resulting number of outcome events per candidate predictor was low, which is acknowledged as a limitation. Analyses were performed in Python 3.13.5 using pandas 2.2.3, NumPy 2.3.5, SciPy 1.17.0, scikit-learn 1.8.0, statsmodels 0.14.6, XGBoost 3.1.3, LightGBM 4.6.0 and SHAP 0.50.0. Descriptive statistics are presented as means ± standard deviation or as counts and percentages, as appropriate.

**Table 1:** DLQI trajectory and 12-month extension by pattern.

Phenotype	Patients, *n*	Baseline	Month 1	Month 3	Month 6	With month-12 data, *n*	Month-12 DLQI	DLQI ≤ 1 at month 12, *n* (%)
Low-burden early control	90	3.99 ± 2.72	2.61 ± 2.53	1.74 ± 2.04	1.47 ± 1.66	57	1.91 ± 2.79	38 (66.7)
High-burden responder	20	15.35 ± 3.45	5.20 ± 3.21	2.90 ± 2.90	1.40 ± 1.60	15	1.80 ± 2.51	9 (60.0)
Persistent burden	23	15.04 ± 7.27	12.61 ± 7.01	11.83 ± 6.97	13.17 ± 5.86	16	7.31 ± 7.79	5 (31.2)

DLQI values are mean ± SD. Patients, *n* is the number of patients assigned to each pattern among the 133 with complete baseline-to-month-6 DLQI. Month-12 values are exploratory and restricted to the 88 clustered patients with an available month-12 measurement. Between-pattern comparison at month 12: Kruskal–Wallis *p* = 0.009 for mean DLQI and chi-square *p* = 0.039 for DLQI ≤ 1. DLQI, Dermatology Life Quality Index; SD, standard deviation.

## Results

### Cohort and longitudinal HRQoL trajectory

The first-stage analytic cohort comprised 219 patients and 831 observations, with 167, 162, 168 and 115 patients contributing DLQI data at months 1, 3, 6 and 12, respectively (Supplementary Table S1). Mean DLQI decreased progressively across follow-up, from 7.78 at baseline to 2.89 at month 12 (Table 2; Figure 1A). In the mixed-effects model, every follow-up visit was significantly lower than baseline (month 1, −2.852 [95% CI −3.628 to −2.076]; month 3, −3.691 [−4.477 to −2.905]; month 6, −3.826 [−4.602 to −3.050]; month 12, −4.771 [−5.657 to −3.885]; all *p* < 0.001; Figure 1B). The proportion of patients with a DLQI of 1 or less rose from 16.9% at baseline to 58.3% at month 12 (Figure 1C).

**Table 2:** Longitudinal DLQI outcomes across follow-up visits.

Visit	Patients, *n*	DLQI, mean ± SD	DLQI ≤ 1, *n* (%)	MCID ≥ 4, *n* (%)
Baseline	219	7.78 ± 7.05	37 (16.9)	—
Month 1	167	4.96 ± 5.51	52 (31.1)	52 (31.1)
Month 3	162	4.28 ± 5.83	79 (48.8)	75 (46.3)
Month 6	168	3.71 ± 5.43	83 (49.4)	70 (41.7)
Month 12	115	2.89 ± 4.44	67 (58.3)	53 (46.1)

Values are mean ± SD or *n* (%). Percentages are calculated among patients with an available observation at each visit. The identical month-1 counts for DLQI ≤ 1 and MCID ≥ 4 were verified in the reconstructed analysis dataset and were coincidental: 52 patients met each endpoint, but only 10 patients met both endpoints. DLQI, Dermatology Life Quality Index; MCID, minimal clinically important difference (≥4-point reduction from baseline); SD, standard deviation. The identical month-1 counts for DLQI ≤ 1 and MCID ≥ 4 (both 52) are coincidental.

**Figure 1:**
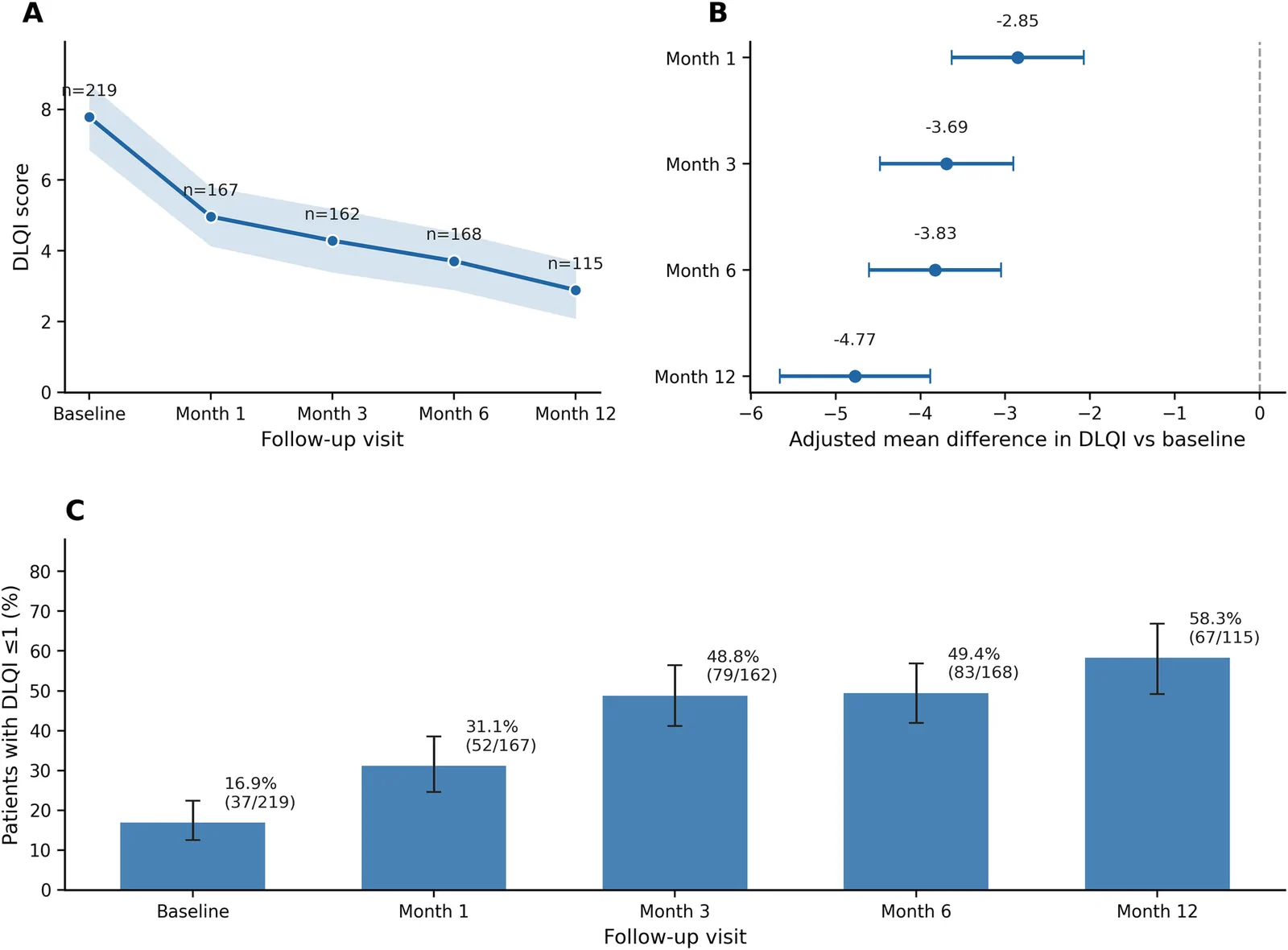
Longitudinal DLQI over 12 months of telepharmacy follow-up. **(A)** Mean DLQI at baseline and at months 1, 3, 6 and 12 with 95% confidence intervals; the number of contributing patients is annotated at each visit. **(B)** Adjusted mean differences in DLQI relative to baseline estimated from the linear mixed-effects model (follow-up visit as a categorical fixed effect, patient as a random intercept), with 95% confidence intervals; the dashed line marks no difference, and every follow-up visit was significantly lower than baseline (all *p* < 0.001). **(C)** Proportion of patients with a DLQI of 1 or less at each visit, with Wilson 95% confidence intervals; counts are shown as n/N. DLQI, Dermatology Life Quality Index.

### Trajectory patterns and their 12-month course

Because a cohort mean can average over patients who follow very different courses, we first characterized the individual DLQI trajectories themselves as our primary analysis. Unsupervised clustering in the 133-patient complete-trajectory cohort resolved three patterns: low-burden early control (n = 90; 67.7%), high-burden responder (*n* = 20; 15.0%) and persistent burden (*n* = 23; 17.3%), with a silhouette of 0.43 and a bootstrap-adjusted Rand index of 0.72. Across the two- to five-cluster Ward solutions the same 23 patients formed a distinct group: they already separated in the two-cluster solution (23 vs. 110) and remained identifiable in the four- and five-cluster solutions, which split only the other segments into smaller subgroups (Supplementary Table S5). The mean trajectories were distinct: both the low-burden and high-burden responder patterns improved to near-remission by month 6, whereas the persistent-burden pattern remained elevated throughout (Table 1). The two high-burden patterns were almost identical at baseline (15.35 ± 3.45 and 15.04 ± 7.27) and separated only from month 1 onward (5.20 ± 3.21 vs. 12.61 ± 7.01). Baseline DLQI differed across patterns overall (*p* < 0.001), a contrast driven by the low-burden pattern; contextual variables did not reach statistical significance; the proportions of obesity (56.5%), drug allergy (30.4%) and age 46–65 years (60.9%) were numerically higher in the persistent-burden pattern but did not differ significantly (Supplementary Table S3). In an exploratory extension among the 88 patients with month-12 data, mean month-12 DLQI was 7.31 ± 7.79 in the persistent-burden pattern vs. 1.91 ± 2.79 and 1.80 ± 2.51 in the low-burden and high-burden responder patterns (Kruskal–Wallis *p* = 0.009), and the proportion reaching a DLQI of 1 or less was 31.2% (5/16) vs. 66.7% (38/57) and 60.0% (9/15) (chi-square *p* = 0.039) (Figure 2).

**Figure 2:**
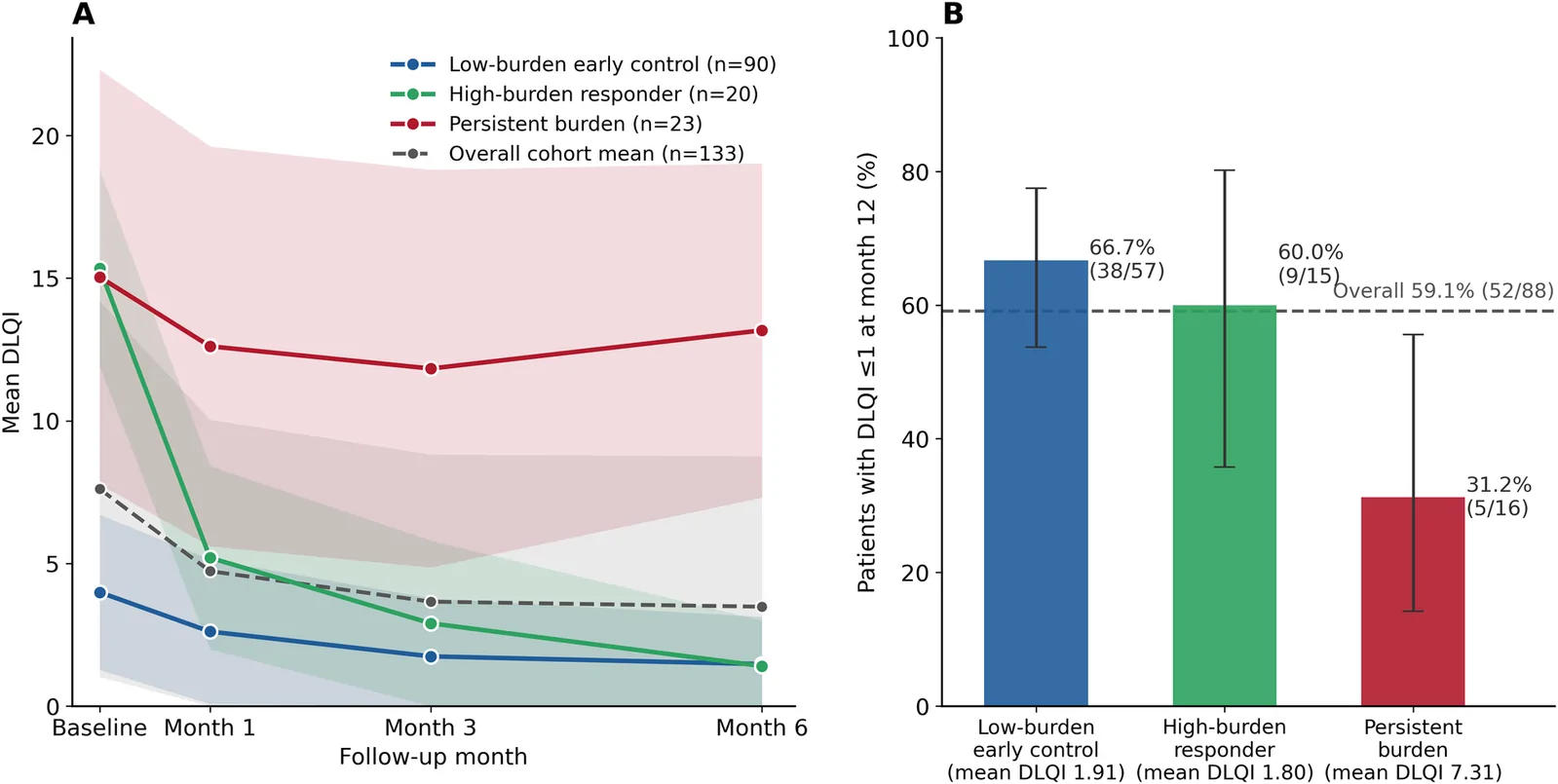
Trajectory patterns and their 12-month course. Unsupervised Ward hierarchical clustering of 6-month DLQI trajectories in 133 patients resolved three exploratory trajectory patterns (data-driven groupings, not validated clinical entities): low-burden early control (*n* = 90), high-burden responder (*n* = 20) and persistent burden (*n* = 23); silhouette 0.43, bootstrap-adjusted Rand index 0.72. **(A)** Observed mean DLQI trajectory for each pattern from baseline to month 6; shaded bands indicate ±1 SD. **(B)** Exploratory month-12 extension: proportion of patients with a DLQI of 1 or less at month 12, with Wilson 95% confidence intervals; the mean month-12 DLQI is shown within each bar. Full estimates and tests are reported in Table 1. DLQI, Dermatology Life Quality Index; SD, standard deviation.

### Benchmarking baseline prediction of clinically meaningful improvement

Identifying a subgroup that remains impaired raises the practical question of whether patients on divergent courses can be flagged at programme entry. As a supporting analysis, we benchmarked prediction of 6-month clinically meaningful improvement, an outcome that, by construction, is tied to baseline DLQI. Although mean DLQI improved across the cohort, fewer than half of the individual patients reached the clinically meaningful threshold: of the 168 patients with a 6-month outcome, 70 (41.7%) achieved a reduction of at least 4 points. Baseline DLQI was higher in those who improved (12.76 ± 6.42) than in those who did not (3.91 ± 4.43). Because this single baseline variable already separated the two groups so markedly, the benchmark ranged from a null rule and a one-predictor baseline-DLQI model to multivariable logistic regression and four ensemble learners; across them, the one-predictor baseline-DLQI logistic model achieved the highest mean AUROC (0.923), matching or exceeding the ensemble models, while the null rule and the multivariable logistic regression performed worse (Table 3). Differences among the best-performing models were small. Among the ensembles, XGBoost showed the best overall balance of ranking precision, balanced classification and probabilistic accuracy (Table 3), and both leading ensembles were well calibrated on averaged out-of-sample probabilities (XGBoost intercept 0.02, slope 0.91, ECE 0.044; LightGBM intercept 0.04, slope 0.90, ECE 0.029) (Figures 3A,B).

**Table 3:** Benchmark of parsimonious and ensemble models for 6-month clinically meaningful improvement (*n* = 168).

Model	AUROC	AUPRC	Balanced accuracy	Brier score
Null rule (baseline DLQI ≥ 4)	0.791 ± 0.047	0.631 ± 0.054	0.791 ± 0.047	0.246 ± 0.055
One-predictor logistic (baseline DLQI)	0.923 ± 0.038	0.855 ± 0.075	0.785 ± 0.068	0.130 ± 0.027
Multivariable logistic regression	0.823 ± 0.058	0.767 ± 0.085	0.728 ± 0.068	0.177 ± 0.036
LightGBM	0.900 ± 0.045	0.830 ± 0.082	0.820 ± 0.054	0.128 ± 0.034
XGBoost	0.903 ± 0.045	0.841 ± 0.083	0.826 ± 0.054	0.127 ± 0.034
RandomForest	0.885 ± 0.059	0.807 ± 0.101	0.832 ± 0.062	0.170 ± 0.015
ExtraTrees	0.780 ± 0.083	0.690 ± 0.097	0.694 ± 0.081	0.215 ± 0.013

Values are mean ± SD across 100 repeated stratified 80/20 train–test splits, computed on held-out test sets. Endpoint: 6-month clinically meaningful improvement (≥4-point DLQI reduction from baseline). Lower Brier scores indicate better probabilistic accuracy. For the deterministic null rule, AUROC equals (sensitivity + specificity)/2 and coincides with its balanced accuracy. AUROC, area under the receiver operating characteristic curve; AUPRC, area under the precision–recall curve; DLQI, Dermatology Life Quality Index; SD, standard deviation.

**Figure 3:**
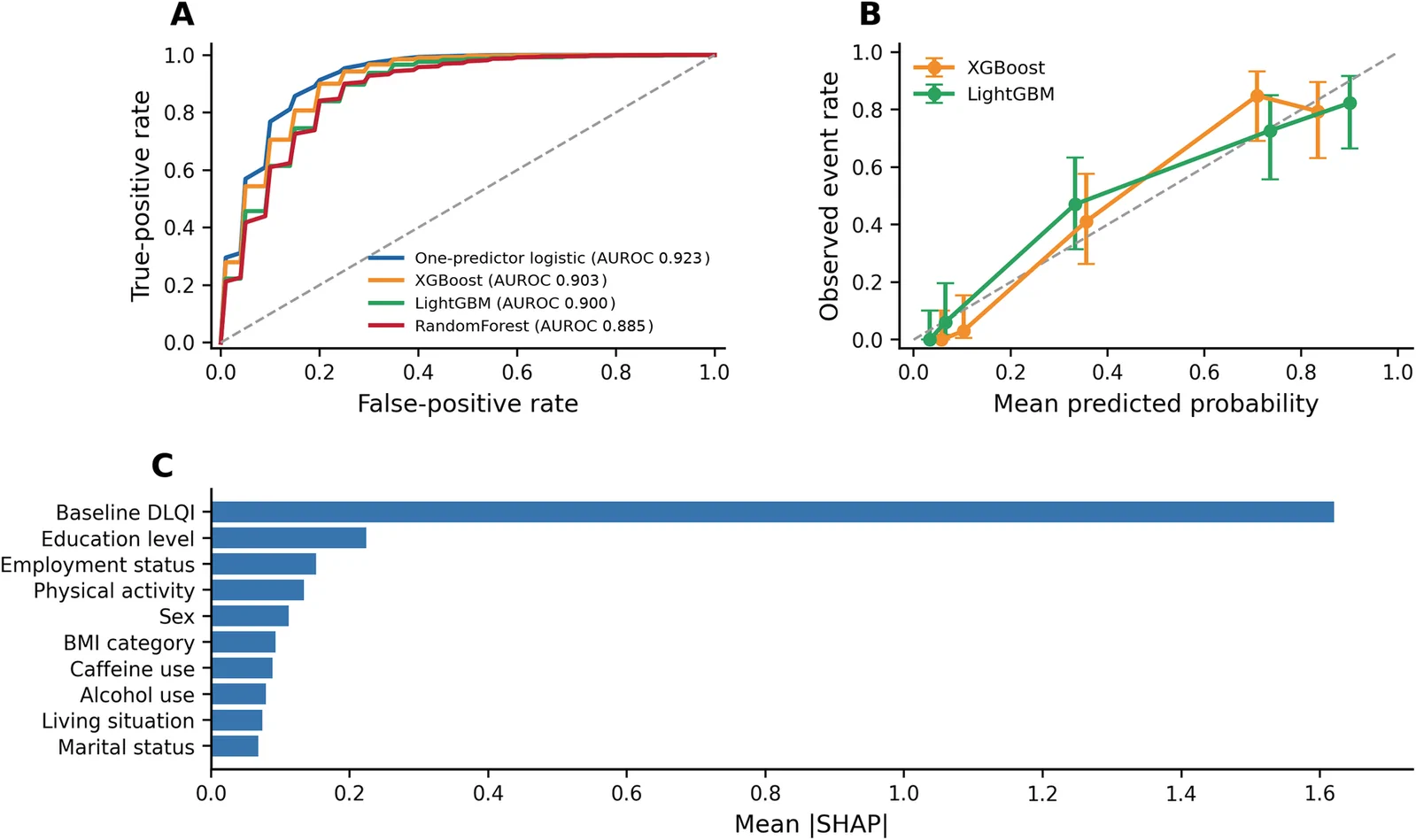
Benchmark of baseline prediction for 6-month clinically meaningful DLQI improvement (≥4-point reduction; *n* = 168). **(A)** Mean receiver operating characteristic (ROC) curves across 100 repeated stratified 80/20 train–test splits for the one-predictor baseline-DLQI logistic model and the leading ensemble models. **(B)** Calibration of the leading ensemble models from per-patient averaged out-of-sample probabilities, summarized with quantile bins and Wilson 95% confidence intervals. **(C)** SHAP importance ranking for the reference XGBoost model; bars represent mean absolute SHAP values. Performance estimates are reported in Table 3. AUROC, area under the ROC curve; DLQI, Dermatology Life Quality Index; ECE, expected calibration error; SHAP, SHapley Additive exPlanations AUROC values shown in the legend correspond to the mean held-out AUROC across the 100 repeated stratified 80/20 partitions reported in Table 3; the displayed ROC curves are averaged curves used for visualization.

To confirm the source of this result, feature contributions in the reference model were examined with SHAP: baseline DLQI dominated the importance ranking, and the remaining features—education level, employment status, physical activity, sex, BMI category, caffeine use, alcohol use, living situation and marital status—followed at markedly lower magnitude (Figure 3C; Supplementary Table S7). In the prespecified baseline-DLQI ≥ 4 subgroup, the one-predictor baseline-DLQI model reached AUROC 0.806 ± 0.092 (AUPRC 0.860 ± 0.071; balanced accuracy 0.739 ± 0.083; Brier score 0.184 ± 0.026). The context-only model, after excluding baseline DLQI and all DLQI-derived quantities, reached AUROC 0.577 ± 0.087 (AUPRC 0.512 ± 0.079; balanced accuracy 0.541 ± 0.075; Brier score 0.252 ± 0.025).

### Updating risk with the first follow-up measurement

The predictions above relied only on information available at programme entry. Because remission (DLQI ≤ 1) is a more demanding target and the first follow-up provides the earliest longitudinal signal in telepharmacy, we examined whether the month-1 measurement improves prediction of remission relative to baseline information. In the same 143-patient dynamic cohort, a baseline-DLQI-only remission model reached AUROC 0.641 ± 0.098 (AUPRC 0.649 ± 0.089; balanced accuracy 0.534 ± 0.083; Brier score 0.241 ± 0.022). The parsimonious month-1 DLQI updating model improved discrimination and probability error, with AUROC 0.753 ± 0.079 (AUPRC 0.727 ± 0.086; balanced accuracy 0.666 ± 0.076; Brier score 0.207 ± 0.027), a calibration intercept of −0.02, a slope of 1.04 and an ECE of 0.021; the broader RandomForest update achieved AUROC 0.682 ± 0.097 (AUPRC 0.683 ± 0.093; balanced accuracy 0.638 ± 0.082; Brier score 0.228 ± 0.030; calibration intercept −0.05, slope 1.05) (Figure 4). Relative to the baseline-only comparator, the month-1 model improved discrimination by a mean of 0.113 AUROC units (95% resampling interval 0.095–0.130).

**Figure 4:**
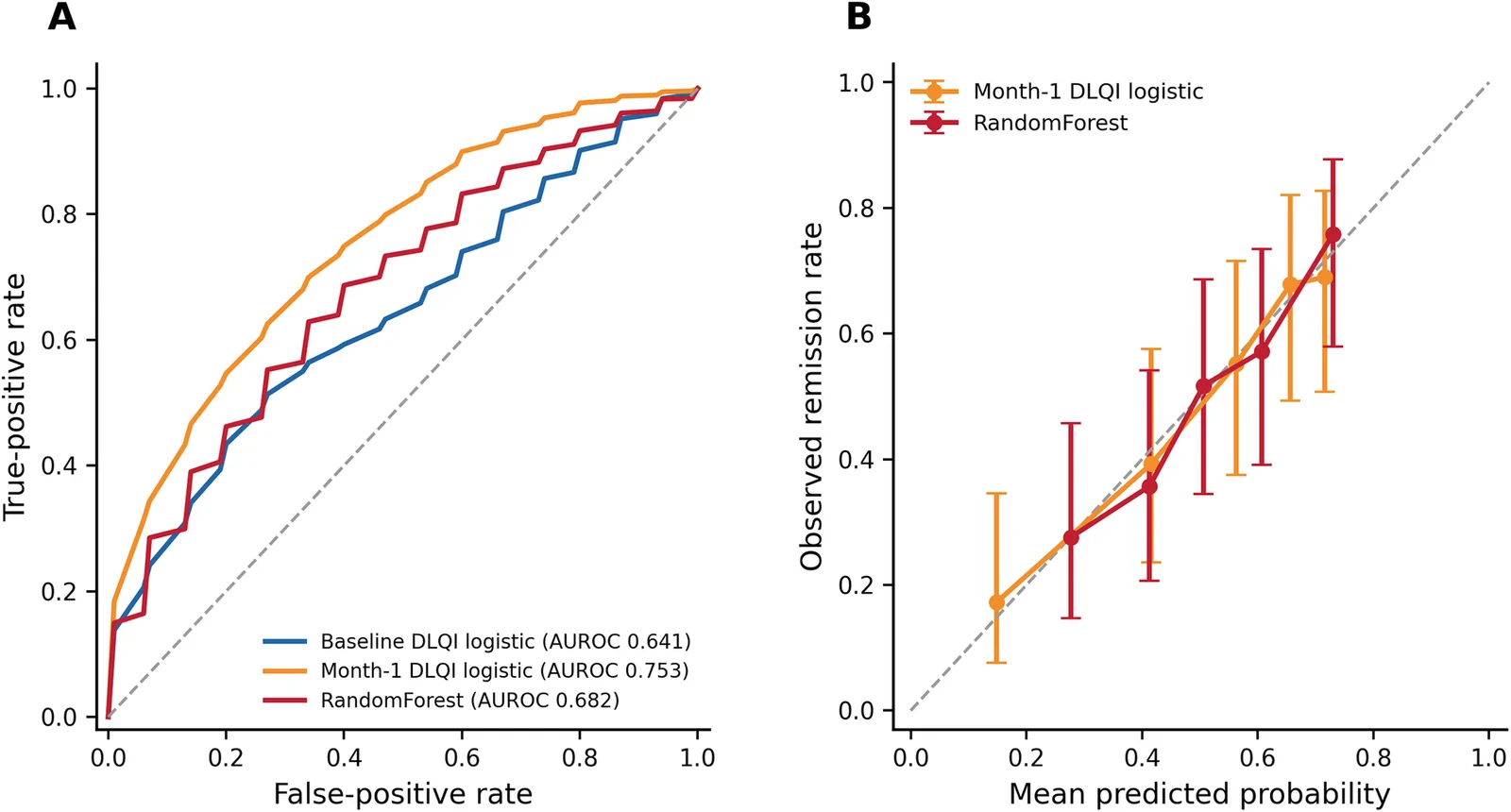
Baseline-only and month-1 updating for 6-month DLQI remission (DLQI ≤ 1; *n* = 143). **(A)** Mean ROC curves across 100 repeated stratified 80/20 train–test splits for the baseline-DLQI-only logistic comparator, the month-1 DLQI logistic-updating model and the RandomForest update. **(B)** Calibration of the month-1 updating model and RandomForest from per-patient averaged out-of-sample probabilities, summarized with quantile bins and Wilson 95% confidence intervals. Performance estimates are reported in the text. AUROC, area under the receiver operating characteristic curve; DLQI, Dermatology Life Quality Index; ECE, expected calibration error.

## Discussion

In this multicenter telepharmacy psoriasis cohort, dermatology-specific HRQoL improved substantially on average over 12 months. This average is incomplete, however, because it conceals substantial between-patient heterogeneity. Unsupervised trajectory-pattern analysis showed that the favourable mean was composed of three distinct courses and that roughly one in six patients, the persistent-burden pattern, remained markedly impaired throughout follow-up and continued to fare worse at 12 months. The clinically relevant question is therefore not how much the cohort improves on average, but whether the minority that does not improve can be identified and managed.

The predictive benchmark clarifies this point. When the outcome is a 4-point DLQI improvement, an endpoint mathematically anchored to baseline severity, a single baseline variable predicted response as well as, or better than, flexible ensemble models, and feature attribution confirmed that baseline DLQI accounted for almost all of the signal. This is consistent with systematic evidence that complex algorithms rarely outperform well-specified regression when benchmarking is rigorous (11), and with reporting standards that call for simple comparators and calibration (12). This does not indicate that machine learning failed; rather, for outcomes governed by baseline burden there is little room for it to add value, and claims of algorithmic superiority in such settings should be interpreted cautiously.

The analyses also compared static baseline information with longitudinal information. On the same 143-patient dynamic cohort and under the same 100 repeated stratified 80/20 framework, a baseline-DLQI-only model predicted 6-month remission with AUROC 0.641 ± 0.098, whereas the month-1 DLQI updating model reached AUROC 0.753 ± 0.079. This paired gain supports the view that the distinctive value of telepharmacy lies not in more elaborate baseline models but in repeated early measurements that allow individual risk to be updated over time. Several non-exclusive mechanisms could account for the additional information carried by the first month, without implying causality: early pharmacological response, treatment adherence and early clinical stabilization may each be partly reflected in the month-1 measurement.

Clinically, these findings point to a concrete use case. The persistent-burden pattern is precisely the group a monitoring programme would want to detect early, and its descriptive association with obesity and greater clinical complexity is coherent with real-world evidence on residual disease burden and biologic switching (13, 14). In practical terms, two DLQI measurements, at programme entry and at the first month, were sufficient to update each patient's probability of 6-month DLQI remission with useful discrimination and good calibration. This early, calibrated estimate could stratify the telepharmacy caseload: patients whose updated risk indicates a favourable course remain on standard remote follow-up, while intensified pharmacotherapeutic follow-up, structured verification of adherence and earlier referral for therapeutic reassessment are concentrated on the minority flagged, as early as month 1, as unlikely to improve. This aligns monitoring intensity with expected need, consistent with the value-based aim of the telepharmacy programme. We emphasize, however, that the patterns and risk estimates are descriptive and internally derived; we did not test, and therefore do not claim, that acting on them changes prognosis. In particular, we defined no risk thresholds and performed no decision-curve or net-benefit analysis. Three elements still require validation: the trajectory patterns, the month-1 updating model with its calibration, and the clinical value of acting on the early-risk signal.

This study has several strengths. It draws on multicenter real-world telepharmacy data, reconstructs a leakage-aware analytic cohort, and integrates unsupervised pattern analysis, benchmarked prediction and dynamic updating within a single coherent framework, with calibration reported alongside discrimination. However, this study also has several limitations that should be acknowledged. It is a secondary analysis of a curated dataset; only the DLQI could be reconstructed across all timepoints, so symptom-specific instruments were unavailable; because the DLQI captures overall quality-of-life impact and does not separate itch, pain, treatment burden or psychosocial load, the patterns describe global HRQoL burden rather than any specific symptom domain; the pattern analysis sample was modest (133 patients, of whom 23 showed persistent burden) and the retained solution showed only moderate separation (silhouette 0.43), an overlap expected because psoriasis recovery is biologically continuous rather than sharply clustered; restricting the pattern analysis cohort to complete baseline-to-month-6 DLQI reduced the sample from 219 to 133 (39% excluded). This reduction was intrinsic to the analysis, since trajectory-pattern analysis necessarily requires complete longitudinal profiles. A baseline comparison between completers and non-completers did not identify statistically significant differences in baseline DLQI or the selected observed covariates (Supplementary Table S6), but selection bias related to differential follow-up cannot be excluded. Most importantly, treatment exposure was largely unrecorded (treatment line and medication-level fields missing in the great majority of records; Supplementary Table S4), so a treatment-informed clustering was not feasible and the persistent-burden pattern cannot be attributed to unmet treatment need, treatment exposure, treatment failure, non-adherence or comorbidity; the study should therefore be read as a monitoring rather than a treatment-response study. The direction of any residual bias is uncertain: if more severely affected patients disengage from questionnaires the persistent-burden pattern would be under-represented, whereas if such patients are retained through closer monitoring it would be over-represented, so its prevalence should be read as an internal, cohort-specific estimate. Under extreme assumptions, if none of the 86 excluded patients followed a persistent-burden course the prevalence of that pattern would fall to 10.5% (23 of 219), whereas if disengagement were concentrated among patients with sustained burden, the direction we consider most plausible, the observed 17.3% would underestimate the true proportion. The supervised improvement and remission models were internally validated only, and the trajectory pattern, being derived by clustering the same DLQI data, should be regarded as exploratory until confirmed in independent cohorts. Estimates for predictors other than baseline DLQI, including the secondary SHAP importances, should be interpreted with caution given the limited number of events per candidate predictor and the potential for overfitting. The patterns are data-driven and do not constitute established clinical entities; the persistent-burden group in particular should be read as hypothesis-generating; their prevalences and boundaries are cohort-specific, and they should not be translated into practice before demonstrating that acting on them improves outcomes. External and prospective validation, ideally incorporating additional clinical and treatment variables, remains the necessary next step. The present objective was methodological benchmarking rather than model deployment.

## Conclusions

In telepharmacy-monitored psoriasis, a favourable average conceals a clinically important minority with persistent HRQoL burden that trajectory-pattern analysis can identify. For outcomes anchored to baseline severity, machine learning adds little beyond a simple baseline-DLQI model; the added value of digital monitoring may instead emerge from longitudinal information, with the first follow-up measurement providing an early, well-calibrated update to individual risk. These findings support a shift from average reporting toward trajectory-based, adaptively updated follow-up: in this setting the added value came not from increasingly complex algorithms but from incorporating early longitudinal information to update individual risk, pending external validation.

## Data Availability

The datasets presented in this article are not readily available because &lt;b&gt;The dataset analysed during the current study is not publicly available because it contains data collected within the NAVETA telepharmacy programme and is subject to data protection and governance restrictions. It is available from the corresponding authors on reasonable request and subject to approval by the data controller. Requests to access the datasets should be directed to salva.herrera@me.com.
