# Supplementary material for: Longitudinal DLQI trajectory patterns and early risk updating in telepharmacy-monitored psoriasis
Source: Front Digit Health. 2026 Oct 26;8:1961097. doi: 10.3389/fdgth.2026.1961097 (PMC13745652; doi:10.3389/fdgth.2026.1961097)
Supplement: Supplementary file 1 [file Table1.docx]

# SUPPLEMENTARY MATERIAL

**Supplementary Table S1. Cohort assembly after duplicate removal and follow-up stage reconstruction.**

| **Stage** | **Count** |
| --- | --- |
| Unique patient–date questionnaire sessions after deduplication | 1,524 |
| Baseline-anchored follow-up stages | 243 |
| First follow-up stages retained for analysis | 219 |
| Total observations in first-stage cohort | 831 |
| Patients with complete baseline-to-month-6 DLQI (trajectory-pattern analysis) | 133 |
| Patients with 6-month outcome (improvement benchmark) | 168 |
| Patients with month-1 and month-6 data (dynamic remission updating) | 143 |
| Patients with 12-month outcome | 115 |

*DLQI, Dermatology Life Quality Index.*

**Supplementary Table S2. Description of the NAVETA psoriasis source cohort.**

| **Characteristic** | **Value** |
| --- | --- |
| Cohort export date | 15 April 2026 (most recent import 13 April 2026) |
| Participating centres, n | 20 |
| Psoriasis source-cohort patients, n | 394 |
| Patients with ≥1 DLQI response, n | 374 |
| DLQI data-collection period | December 2021 – March 2026 |
| Age, years, median (IQR) | 50 (40–59) |
| Age, years, range | 18–83 |
| Women, n (%) | 190 (48.2) |
| Men, n (%) | 176 (44.7) |
| Sex not recorded, n (%) | 28 (7.1) |
| Patients per centre, median (range) | 11 (1–119) |
| Analytic cohort (first follow-up stage per patient), n | 219 |

*Values describe the psoriasis source cohort from the NAVETA telepharmacy programme (394 patients). The analytic cohort used for all analyses (first follow-up stage per patient, complete-case timepoints; n = 219) is a subset of this source cohort, whose assembly is detailed in Supplementary Table S1. Sex percentages are of the 394 source-cohort patients; age was available for 364. DLQI, Dermatology Life Quality Index; IQR, interquartile range.*

**Supplementary Table S3. Baseline characteristics of the 133-patient trajectory-pattern cohort, by pattern.**

| **Characteristic** | **Low-burden early control (n = 90)** | **High-burden responder (n = 20)** | **Persistent burden (n = 23)** | **P value** |
| --- | --- | --- | --- | --- |
| Baseline DLQI, mean (SD) | 3.99 (2.72) | 15.35 (3.45) | 15.04 (7.27) | <0.001 |
| Female sex, n (%) | 45 (50.0) | 12 (60.0) | 12 (52.2) | 0.733 |
| Age 46–65 years, n (%) | 46 (51.1) | 5 (25.0) | 14 (60.9) | 0.068 |
| Obesity, n (%) | 25 (27.8) | 5 (25.0) | 13 (56.5) | 0.073 |
| Currently employed, n (%) | 62 (68.9) | 13 (65.0) | 14 (60.9) | 0.105 |
| University degree, n (%) | 22 (24.4) | 7 (35.0) | 4 (17.4) | 0.114 |
| Living with family, n (%) | 44 (48.9) | 7 (35.0) | 12 (52.2) | 0.702 |
| Drug allergy, n (%) | 16 (17.8) | 3 (15.0) | 7 (30.4) | 0.337 |

*Values are mean (SD) or n (%). P values are descriptive, from Kruskal–Wallis tests (continuous variables) or chi-square tests (categorical variables). DLQI, Dermatology Life Quality Index; SD, standard deviation.*

**Supplementary Table S4. Baseline predictor missingness and model inclusion.**

| **Variable** | **Role** | **Primary benchmark n=168 missing, n (%)** | **Dynamic cohort n=143 missing, n (%)** |
| --- | --- | --- | --- |
| Baseline DLQI | Included in baseline models | 0 (0.0) | 0 (0.0) |
| Sex | Included in baseline models | 3 (1.8) | 2 (1.4) |
| Age range | Included in baseline models | 6 (3.6) | 5 (3.5) |
| BMI range | Included in baseline models | 0 (0.0) | 0 (0.0) |
| Physical activity | Included in baseline models | 41 (24.4) | 35 (24.5) |
| Balanced diet | Included in baseline models | 41 (24.4) | 35 (24.5) |
| Patient-association membership | Included in baseline models | 1 (0.6) | 1 (0.7) |
| Marital status | Included in baseline models | 4 (2.4) | 4 (2.8) |
| Education level | Included in baseline models | 1 (0.6) | 1 (0.7) |
| Employment status | Included in baseline models | 1 (0.6) | 1 (0.7) |
| Living situation | Included in baseline models | 4 (2.4) | 4 (2.8) |
| Alcohol use | Included in baseline models | 2 (1.2) | 2 (1.4) |
| Tobacco use | Included in baseline models | 41 (24.4) | 35 (24.5) |
| Caffeine use | Included in baseline models | 41 (24.4) | 35 (24.5) |
| Other substance use | Included in baseline models | 2 (1.2) | 2 (1.4) |
| Drug allergy | Included in baseline models | 0 (0.0) | 0 (0.0) |
| Age at smoking initiation | Excluded from primary modelling | 86 (51.2) | 75 (52.4) |
| Diagnosis date | Excluded from primary modelling | 123 (73.2) | 105 (73.4) |
| Treatment line | Excluded from primary modelling | 167 (99.4) | 142 (99.3) |
| Current medication | Excluded from primary modelling | 123 (73.2) | 105 (73.4) |

*Baseline DLQI was the only predictor in the null rule and one-predictor improvement model. Multivariable logistic regression and ensemble models used baseline DLQI plus the included baseline covariates. The context-only sensitivity model removed baseline DLQI and DLQI-derived quantities. DLQI outcomes and follow-up DLQI values used to define endpoints were not imputed.*

**Supplementary Table S5. Internal indices for alternative Ward clustering solutions.**

| **k** | **Cluster sizes** | **Silhouette** | **Calinski–Harabasz** | **Davies–Bouldin** |
| --- | --- | --- | --- | --- |
| 2 | 23; 110 | 0.563 | 115.2 | 0.874 |
| 3 | 23; 20; 90 | 0.428 | 99.1 | 0.965 |
| 4 | 3; 20; 20; 90 | 0.445 | 106.6 | 0.905 |
| 5 | 3; 7; 13; 20; 90 | 0.455 | 100.2 | 0.950 |

*The three-cluster solution was retained because it separated a clinically interpretable persistent-burden pattern while preserving a parsimonious pattern structure.*

**Supplementary Table S6. Baseline comparison of trajectory completers and non-completers.**

| **Characteristic** | **Completers (n=133)** | **Non-completers (n=86)** | **P value** |
| --- | --- | --- | --- |
| Baseline DLQI, mean (SD) | 7.61 (6.57) | 8.05 (7.77) | 0.910 |
| Female sex, n (%) | 69 (51.9) | 46 (53.5) | 0.816 |
| Age 46–65 years, n (%) | 65 (48.9) | 38 (44.2) | 0.497 |
| Obesity, n (%) | 43 (32.3) | 31 (36.0) | 0.570 |
| Currently employed, n (%) | 89 (66.9) | 47 (54.7) | 0.068 |
| University degree, n (%) | 33 (24.8) | 13 (15.1) | 0.085 |
| Living with family, n (%) | 63 (47.4) | 40 (46.5) | 0.901 |
| Drug allergy, n (%) | 26 (19.5) | 17 (19.8) | 0.968 |

*Completers were patients with complete baseline, month-1, month-3 and month-6 DLQI data and therefore eligible for trajectory-pattern analysis. P values are descriptive, from Kruskal–Wallis tests for continuous variables and chi-square tests for categorical variables. DLQI, Dermatology Life Quality Index.*

**Supplementary Table S7.** SHAP feature importance (mean|SHAP|) for the reference XGBoost model.

| **Feature** | **mean\|SHAP\|** |
| --- | --- |
| Baseline DLQI | 1.620 |
| Education level | 0.224 |
| Employment status | 0.152 |
| Physical activity | 0.134 |
| Sex | 0.112 |
| BMI category | 0.093 |
| Caffeine use | 0.089 |
| Alcohol use | 0.079 |
| Living situation | 0.074 |
| Marital status | 0.069 |
| Age category | 0.045 |
| Tobacco use | 0.037 |
| Balanced diet | 0.016 |
| Drug allergy | 0.008 |
| Patient association | 0.000 |
| Other substance use | 0.000 |

Mean absolute SHAP values for the reference XGBoost model of 6-month clinically meaningful improvement, ranked in decreasing order of importance; these values correspond to the bars displayed in Figure 3C. SHAP, SHapley Additive exPlanations.
